# Mineralized Remains as Adjacent Proxy for Radiocarbon Dating

**DOI:** 10.1021/acs.analchem.5c03812

**Published:** 2026-01-14

**Authors:** Laura Hendriks, Clémence Iacconi, Luc Robbiola, Elsa Desplanques, Negar Haghipour, Corentin Reynaud, Loïc Bertrand

**Affiliations:** † HEIA School of Engineering and Architecture of Fribourg, 128872 HES-SO University of Applied Sciences and Arts Western Switzerland, Pérolles 80, Fribourg 1700, Switzerland; ‡ 27048 Université Paris-Saclay, ENS Paris-Saclay, CNRS, PPSM, 4 avenue des sciences, Gif-sur-Yvette 91190, France; § 131868 TRACES, CNRS, ministère de la Culture, Université Toulouse Jean Jaurès, UMR 5608, Toulouse 31000, France; ∥ 27063 Sorbonne Université, Centre André Chastel, 2 rue Vivienne, Paris 75002, France; ⊥ Laboratory of Ion Beam Physics, ETH Zurich, Otto-Stern-Weg 5, Zurich 8093, Switzerland; # Geological Institute, ETH Zurich, Sonnegstrasse 5, Zurich 8092, Switzerland

## Abstract

Since the 1950s, radiocarbon dating of archeological remains has evolved significantly with the advent of new instruments, protocols, and redesigned concepts. Here, we show that the recovery of chronological information “stored” locally can be achieved by the selective dating of carbonates present in adjacent mineralized organic materials. We present results from the iconic Iron Age site of Creney-le-Paradis (Aube, France). The ^14^C ages extracted using an innovative selective strategy provide new evidence for the chronology of the foundation of the site. We show that the copper carbonate accretions retained the signature of an anthropogenic humus layer, accurately dated between 808 and 790 BC, allowing us to infer human activity associated with the foundation of the burial mound. This work opens the way for the development of spatially resolved dating imagery within sites, where the analysis of series of microsamples could document the chronology of their formation.

## Graphical abstract

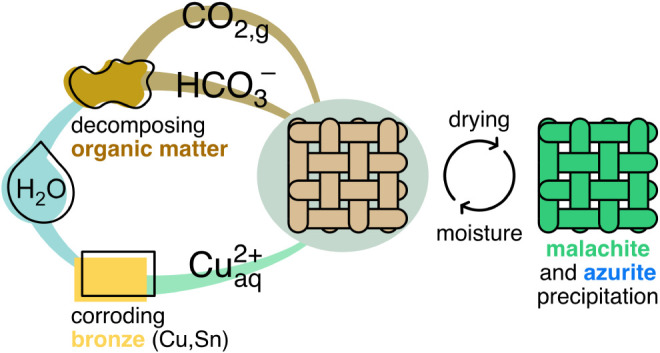

## Introduction

In order to place a discovery within a broader historical framework, archeologists rely on relative or absolute dating. The former is often based on a stylistic comparison to assess contemporaneity, while the latter provides a specific calendar window based on a measurement. Among other techniques, radiocarbon (^14^C) dating has considerably enriched our understanding of the temporality of past events. In recent years, methodological and technological advancement has not only revolutionized sample size requirement (from grams to μg of carbon), but also transformed our way of analyzing the ^14^C content of a sample beyond recognition from β-decay to measurement of single ^14^C atoms.^1^ Coupling with Bayesian chronological modeling has become indispensable in establishing chronologies in many research fields.^2^ Notable archeological examples include dating of the popular alpine Iceman, Ötzi^3^ or of the largely debated Shroud of Turin.^4,5^ Direct ^14^C dating of lipids extracted from organic residues found in pottery vessels has largely renewed the field of archeological dietary studies, allowing to trace the evolution of lactase persistence in Europe.^6,7^ As long as organic material can be extracted, ^14^C dating is a universal technique capable of dating the last 45,000 years of human history. Over recent years, advanced characterization techniques have been used to better understand the fundamental mechanisms governing the mineralization of organic remains in archeological contexts, thereby demonstrating the need to establish a clearer link between the organic and inorganic fractions in these samples.^8–10^ We aim at combining these techniques with ^14^C dating methods, which have so far proven insufficient on their own for dating mineralized textiles.^11^

In this work, we report a paradigm shift, as we develop a specific protocol to recover the ^14^C signature stored in mineralized organic remains. We elucidate the authigenesis of the neoformed carbonates that led to the exceptional state of structural preservation of the mineralized textile fragments; of particular concern was determining the origin of the carbon atoms. We demonstrate that mineralized remains are capable of storing the ^14^C signature of human activities associated with the foundation of the burial mound from the major Iron Age burial site of Creney-le-Paradis (Aube, France).

### Archeological Context

Creney-le-Paradis has recently been confirmed as one of the most important Iron Age burial sites in Western Europe in terms of status, based on indirect evidence placing it on a par with the contemporary elite sites of Lavau and Vix.^12^ Excavations at the site in the late 1980s identified two main structures, a necropolis used from the final Bronze Age to the beginning of the Late Iron Age (eighth–fifth century BC), and an indigenous farm, associated with a far later Gallo–Roman occupation (52 BC–486 AD).^13^ Excavations at the necropolis revealed three phases of concentrically superimposed construction of a tumulus with a large central burial chamber (ca. 3 m × 2 m), surrounded by vertical planks forming a formwork dug into a chalky soil, partially covered with organic earth slabs topped by a cap of limestone fragments (Supporting Information Figure S1).^13^

However, contextual and material information is extremely sparse: (1) the site was explored, reportedly at the end of the 19th century, leaving only scattered information about the original layout of the archeological remains,^13^ (2) cultivation and associated plowing leveled out much of the site structure, and (3) finally, the site was entirely destroyed to make way for the extension of a freeway access boulevard, after which most of the archeological material was dispersed. No direct dating was attempted before destruction. The chronology of the site was proposed based on stylistic features and comparison with neighboring sites.^13^ The central pit is thought to date from the late sixth or fifth century BC.^12,13^

Therein, 99 bronze-based fragments covered with textile remains were identified ranging in size from a few millimeters to a few centimeters^12^ (Supporting Information Section S2.1). These woolen textile finds were preserved owing to a form of exceptional organic conservation, designated as mineralization.^8,14,15^ Mineralization occurs when organic materials are closely associated with corroding metal artifacts, as in burials, mines or, more rarely, domestic contexts.^16^

The main mineral phases identified here are the basic copper carbonates, malachite Cu_2_(OH)_2_CO_3_ and azurite Cu_3_(OH)_2_(CO_3_)_2_ (SEM, SR-XRD; Supporting Information Figure S3), which show color variations from green to light or dark blue (Figure 1).

**Figure 1:**
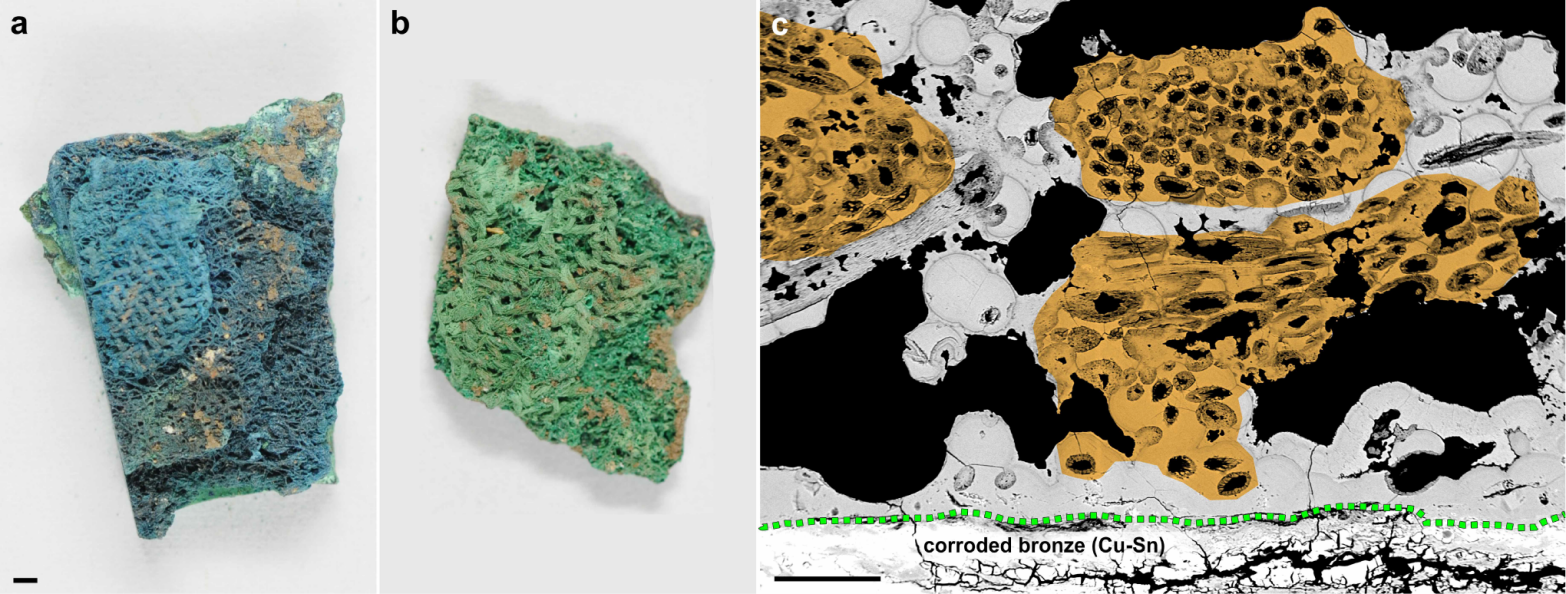
Fragments of mineralized wool textiles from Creney-le-Paradis (Aube, France). (a,b) Macrophotographs show the two main categories of corrosion products encountered in textile fragments: azurite (a) and malachite (b); scale bar: 1 mm. (c) Backscattered electron (BSE) image of mineralized textile fibers on a corroded bronze sheet. Altered longitudinal and transversal textile fibers (orange areas), embedded in Cu­(II) carbonate corrosion deposits (gray areas), are linked to uniform corrosion of the bronze with a preserved limit of the original surface (green dashed line). Pseudomorphic fibrils are clearly visible in each fiber; scale bar: 100 μm.

## Experimental Section

blue-colored solution typical for the hexaaquacopper

### Material Characterization

Optical examination of the mineralized textile fragments was carried out using a stereomicroscope (AxioZoom V16, Zeiss) with magnifications of up to 56× at the TRACES Laboratory (Toulouse, France). Additionally, cross-sections were studied under polarized light, in bright and dark field modes with magnifications from 50× to 1000×. Due to their composition, which is mainly based on corroded copper compounds, none of the samples was treated by metalization, i.e., no conductive film (carbon or gold deposited) was applied before analysis, in order to assess whether there were any variations in the carbon (and nitrogen) composition.

*Scanning electron microscopy–energy dispersive spectroscopy (SEM-EDS)* under variable pressure was performed on the scanning electron microscope at the TRACES Laboratory (EVO 25 LaB6 VP, Zeiss) for three mineralized textile fragments (samples N7, N8 and N12) and two cross sections (N7 and N12). Secondary emission (SE) and backscattered emission (BSE) images were collected on all samples (voltage: 10–15 kV; VP: 30 Pa; beam intensity: 150–450 nA; working distance: 9.4–9.6 mm). Elemental X-ray microanalysis and mapping (1 h / 6 kcps) were carried out on all samples (voltage: 20 kV; working distance: 9.4–9.8 mm) with an EDS system (Quantax 200 with SDD XFlash 6/30 detector, Esprit 2.1 Software, Bruker).

*Synchrotron X-ray diffraction (SR-XRD)* patterns were collected for three fiber samples: A1_4, A1_5 and A2A3_3 mounted vertically on a metal support at an energy of 20 keV to maximize their transmission (acquisition time: 1 s/point; beam diameter: 10 μm), at the PUMA beamline of the SOLEIL synchrotron facility, France.^17^ The 2D XRD patterns were integrated azimuthally using the PyFAI software package.^18^ Diffractogram peaks were indexed using the International Centre for Diffraction Data (ICDD) PDF4+ database.^19^

### Selective Thermal Sample Preparation Strategy

The thermal behavior of copper carbonates was first investigated by thermogravimetric analysis (TGA) on a TGA/DSC instrument (Mettler Toledo AG, Greifensee, Switzerland) in order to determine the optimal parameters for the thermal decomposition of copper carbonates. In practice, the conversion of the samples to CO_2_ was performed in sealed quartz tubes under vacuum (*l* = 16 cm, 0.8 cm diameter; Möller, Switzerland), heated to 643 K for 30 min in a muffle furnace (SOLO Industrieöfen GmbH, Biel, Switzerland) (Figure 2a–e). Through cryo-trapping, the produced CO_2_ was transferred to Pyrex tubes (*l* = 7 cm, 0.4 cm diameter; Möller, Switzerland). The induced thermal decomposition of mineralized textile samples produces gaseous CO_2_, H_2_O, and a black solid residue composed of CuO and organic matter, if any (Figure 2f). Parallel processing of the gas and solid phases enabled the final selective retrieval of the ^14^C signature stored both in the carbonate anion, i.e., the mineralized fraction, and in the organic fraction. After the thermal decomposition process, CO_2_ was purified in a dedicated vacuum line for cryo-trapping. The copresence of water vapor was shown to have a negative impact on the ionization of CO_2_ in the gas ion source for gaseous ^14^C measurements^20^, thus its removal is critical. As gases are released by breaking the glass ampule (Figure 2f), water is trapped in a Peltier module cooled to 248 K, while carbon dioxide is trapped in a calibrated volume coldfinger fitted with a pressure sensor and cooled to 78 K with liquid nitrogen (Figure 2g). Upon removal of the cooling trap, CO_2_ expands and equilibrates with ambient temperature, allowing its manometrical quantification. The latter was finally transferred, frozen, and flame-sealed in a borosilicate tube cooled with a liquid nitrogen trap. ^14^C measurement of the purified CO_2_ fraction, corresponding to the mineral carbon in the carbonates, was carried out via a gas interface system (GIS) coupled to the AMS (Figure 2l,n). The black solid residue left in the broken glass ampule (mainly made of CuO and organic carbon, if any) was treated with 1-M HCl (Figure 2h–j), ensuring the removal of other carbonate contamination (i.e., calcite from the burial environment) whose dissociation temperatures are higher than copper carbonates (>643 K). The dried solid residue was packed in an aluminum boat and its ^14^C content measured by direct combustion in an elemental analyzer (EA) coupled to the AMS (Figure 2m,n).

**Figure 2:**
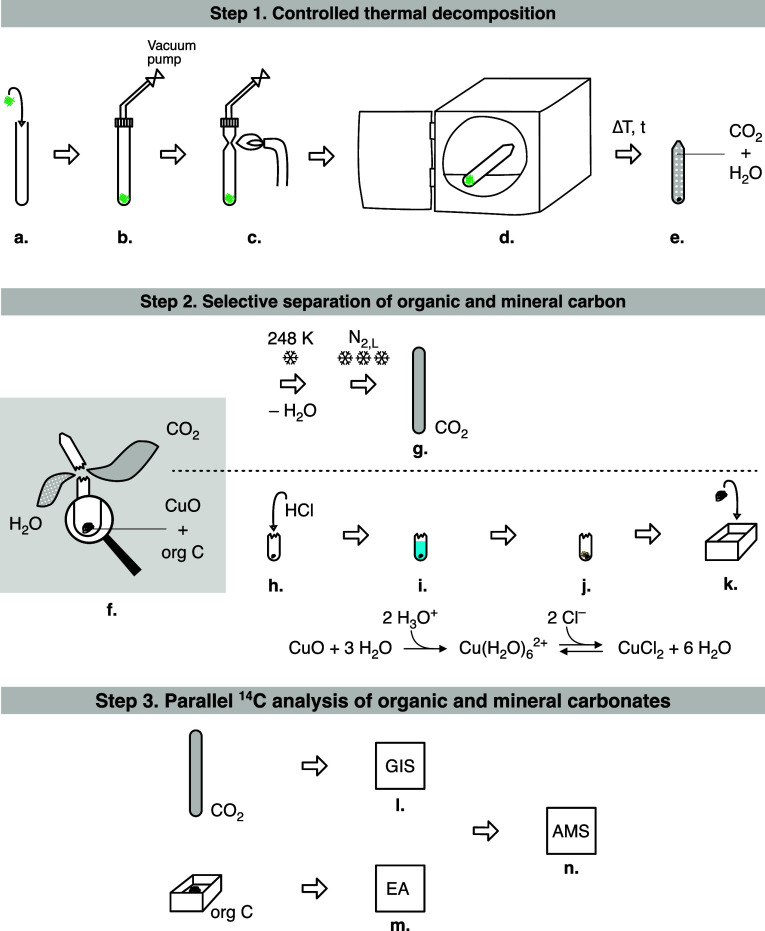
Procedure developed for the radiocarbon dating of basic copper carbonates. The sample is inserted into a prebaked quartz tube (a), plugged on a vacuum line (b) and sealed under vacuum (c). The enclosed sample is heated to 643 K for 30 min (d) in a muffle furnace, resulting in the thermal dissociation of the basic copper carbonates (e). After the thermal decomposition procedure, the glass ampule is broken (f) to retrieve the frozen CO_2_, which is transferred to a borosilicate tube following cryo-trapping (g). Any remaining solid material is washed with 1-M HCl (h), forming a blue-colored solution typical for the hexaaquacopper­(II) ion,  [Cu­(H_2_O)_6_]^2+^ (i), which is dried down (j) and finally wrapped in an aluminum boat (k). The carbonate fraction and organic matter are measured on an AMS. The borosilicate tube containing the CO_2_ is cracked and via the gas ion source interface (GIS) transferred to the AMS (l,n) while the packed organic matter is combusted in an elemental analyzer (EA) and the resulting CO_2_ is transferred to the AMS (m,n).

### Radiocarbon Analysis

All radiocarbon measurements were performed on the compact Mini Carbon Dating System (MICADAS) at the Laboratory of Ion Beam Physics, ETH Zurich, Switzerland.^21,22^ Standard normalization and blank correction were performed using the BATS data reduction program^23^ while samples bearing less than 40 μg C were additionally corrected following the model of constant contamination.^24^ Correction parameters for the cracker setup were a carbon mass of 1.6 ± 0.4 μg with a F^14^C of 0.37 ± 0.05, while for the EA-AMS setup, it was a carbon mass of 0.8 ± 0.2 μg with a F^14^C of 0.50 ± 0.08. Radiocarbon ages were calibrated using Oxcal v.4.4 software with the Intcal20 atmospheric curve.^25–27^

### Stable Carbon Isotope Analysis

Stable carbon isotope ratios were analyzed on an elemental analyzer (Vario MICROcube, Elementar) coupled to an isotope ratio mass spectrometer (visION, Isoprime). The measured ^13^C/^12^C stable isotope ratios are reported as δ^13^C in standard part per thousands (‰) units^28^ with an accuracy better than ± 0.1‰ to trace the source apportionment of the carbon.

## Results and Discussion

### Description of the Mineralization Facies

A high degree of homogeneity in the corrosion copper deposits and mineralized fibers was observed among all the fragments studied. The textiles showed significant loss of cohesion and nearly complete dispersion of organic matter, which would have appeared darker in BSE images if preserved (Figure 3). Two primary patterns emerged. First, copper corrosion products sometimes coat fibers as a fine “gangue,” creating a porous network (Figure 3a-b). Second, mineralized fibers can be fully embedded within copper deposits, exhibiting the same chemical contrast as copper hydroxide carbonate minerals (Figure 3c).

**Figure 3:**
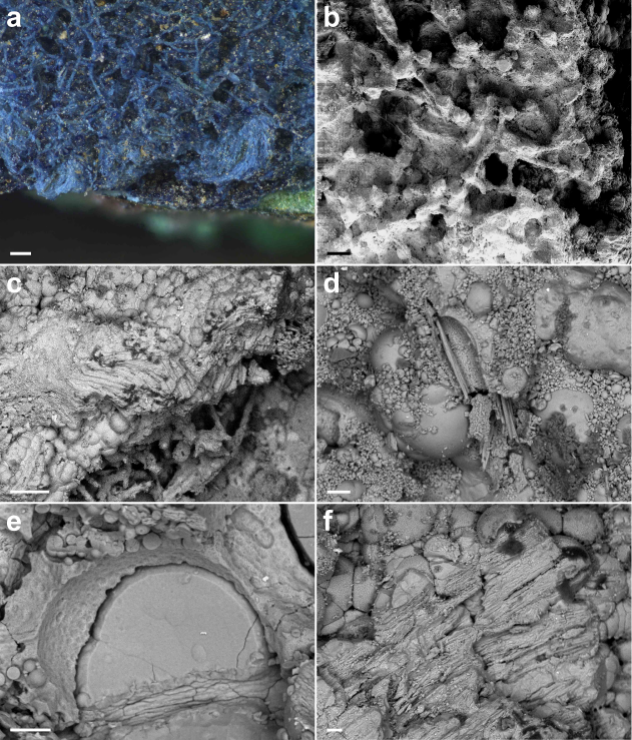
Mineralization facies. Optical microscope image of the mineralized textile fragment N12 (a). BSE images of mineralized textile fragment N12 (b,e) and N7 (c,d,f). Scale bars in (a–c): 200 μm and in (d–f): 20 μm.

At larger scales, fiber alteration takes several forms. Fibers may retain their shape within a mineralized matrix while losing either their central cortex (Figure 3d) or being filled with copper corrosion products (Figure 3e-f). After fiber dissolution, the resulting void can be filled with mineral crystals forming a compact cylindrical pseudomorphic volume (Figure 3e). Alternatively, fibers may fracture and lose their surface structure, revealing residual internal mineralization (Figure 3f). These distinct degradation modes likely reflect varying mineralization kinetics driven by local environmental conditions, including fragment position and aqueous electrolyte composition.

### Selective Thermal Sample Preparation Strategy

Standard ^14^C protocols involve alternating washes in acidic, alkaline, and acidic solution. These approaches target organic matter and remove contamination by inorganic carbonates. The resulting contaminant-free organic fraction is converted to CO_2_ after high-temperature (1223 K) combustion with an oxidizing agent. Metal carbonate accretions are dissolved by this process and eliminated (eqs. 1 and 2, Scheme 1). However, during the mineralization of keratin-based materials, little or no organic carbon remains for ^14^C dating, which is attributed to cleavage of the protein backbone, while the most labile polypeptides are leached out.

**Scheme 1:**
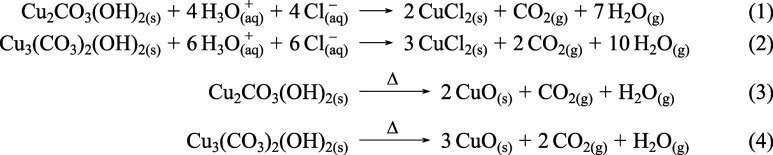
(1, 2) Traditional ^14^C Protocols Involving Washing the Dated Sample with a Solution of Hydrochloric Acid; (3,4) Thermal Decomposition of Malachite and Azurite^a^

a In presence of hydroxychloric acide, malachite and azurite react to form copper chloride, carbon dioxide, and water, which are all eliminated in traditional ^14^C dating protocols (1,2). Here, we optimized the thermal decomposition of malachite (3) and azurite (4) to selectively isolate and date the mineral carbon from the carbon dioxide produced.

For this work, we departed from this traditional approach and tested whether the CO_2_ released from the carbonate during acid hydrolysis (and normally eliminated) retained any chronological information. We developed a protocol targeting selectively the ^14^C signature stored in the copper­(II) hydroxide carbonate anion. Both azurite and malachite readily dissociate above 498 K to give copper­(II) oxide (CuO) and generated gases, CO_2_ and H_2_O.^29^ While this reaction is characterized by multiple overlapping steps depending on mineralogy and environmental conditions (grain size, heating rate, atmospheric pressure, and composition),^29,30^ we developed a protocol to isolate carbon dioxide in a single smooth step, indicative of a full dissociation of carbonates and conversion to carbon dioxide (eqs. 3 and 4, Scheme 1; Figure 4).

**Figure 4:**
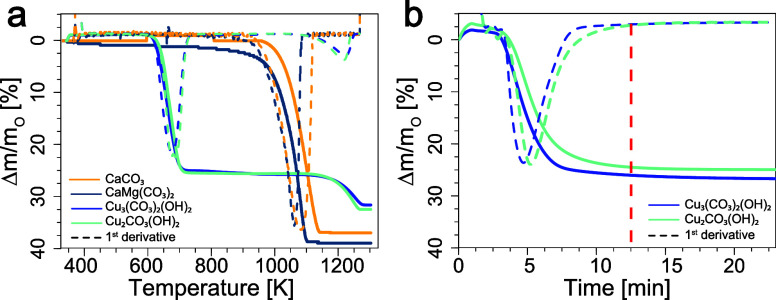
Comparison of the thermal decomposition of four carbonate species by thermogravimetric analysis. (a) In a N_2_ atmosphere, the thermal decomposition of calcite (CaCO_3_, gold), dolomite (CaMg­(CO_3_)_2_, dark blue), azurite (Cu_3_(OH)_2_(CO_3_)_2_, blue) and malachite (Cu_2_(OH)_2_CO_3_, cyan) was carried out at a constant heating rate of 306 K/min from room temperature to 1273 K. (b) The corresponding isothermal analysis (643 K for 2 h) indicates that both copper­(II) hydroxide carbonates decompose into carbon dioxide and water under the given conditions in a single step.

Both copper­(II) hydroxide carbonates exhibited similar decomposition behavior when heated at a constant rate of 306 K/min from room temperature to 1273 K in a N_2_ atmosphere (Figure 4a). A strong endothermic peak was observed at ca. 643 K, attributed to the simultaneous loss of water and CO_2_, followed by a second minor peak above 1273 K, associated with the reduction of CuO to Cu_2_O. The exact temperature range differed slightly between the two mineral phases. For malachite, the decomposition reaction started ca. 20 K below that of azurite, in accordance with Kiseleva et al.^31^ The corresponding isothermal analysis run at 643 K revealed that the reaction was complete within 10 min (Figure 4b). The proposed approach is inherently selective. Despite a chalk-rich context, no interference from other other soil/groundwater carbonates was observed in the measured carbon signal. Calcite and dolomite are commonly present in soil and are major constituents of rocks such as limestone and marble. However, as shown in the TGA data (Figure 4a), their decomposition occurs at significantly higher temperatures (>1000 K) than those applied here, preventing the release of CO_2_ from these geological carbonates.

The specific ^14^C signature of both copper­(II) hydroxide carbonates could be isolated in a single step after heating the samples at 643 K for 30 min, with an extra margin to mitigate any possible thermal inertia of the muffle furnace. The efficiency of the proposed approach is given by the carbon recovery yield (Radiocarbon dating; Supporting Information Table S1). Generally, a 94% recovery rate between the theoretical and the obtained value was observed, with lower yields for partially mineralized samples.

### Radiocarbon Analysis of Carbonate Accretions

A total of six samples were analyzed, all consisting of fibers extracted from mineralized textiles in contact with the metal substrate (Supporting Information Table S2). All samples showed a high degree of mineralization, leaving little hope as to the significant presence of remaining organic matter. The new protocol developed here enabled careful selective dating of both organic and mineral carbon using ^14^C AMS. As previously mentioned, gaseous CO_2_, H_2_O, and a black solid residue composed of CuO and organic matter are produced by thermal decomposition of mineralized textiles. Thus, the sequential processing of the gas and solid phases enabled the final selective retrieval of the ^14^C signature stored both in the carbonate anion, i.e., the mineralized fraction, and in the organic fraction. Only one of the samples yielded >2 μg of organic carbon (sample A1_6, ca. 50 μg of carbon). This low amount of carbon translated into a large chronological window, 795–412 BC (95.4% probability range), covering the entire Early Iron Age and the beginning of the Late Iron Age (Figure 5a). This confirms the difficulty of directly dating mineralized matter using traditional ^14^C approaches due to the absence or very low residual organic carbon content.^11^

**Figure 5:**
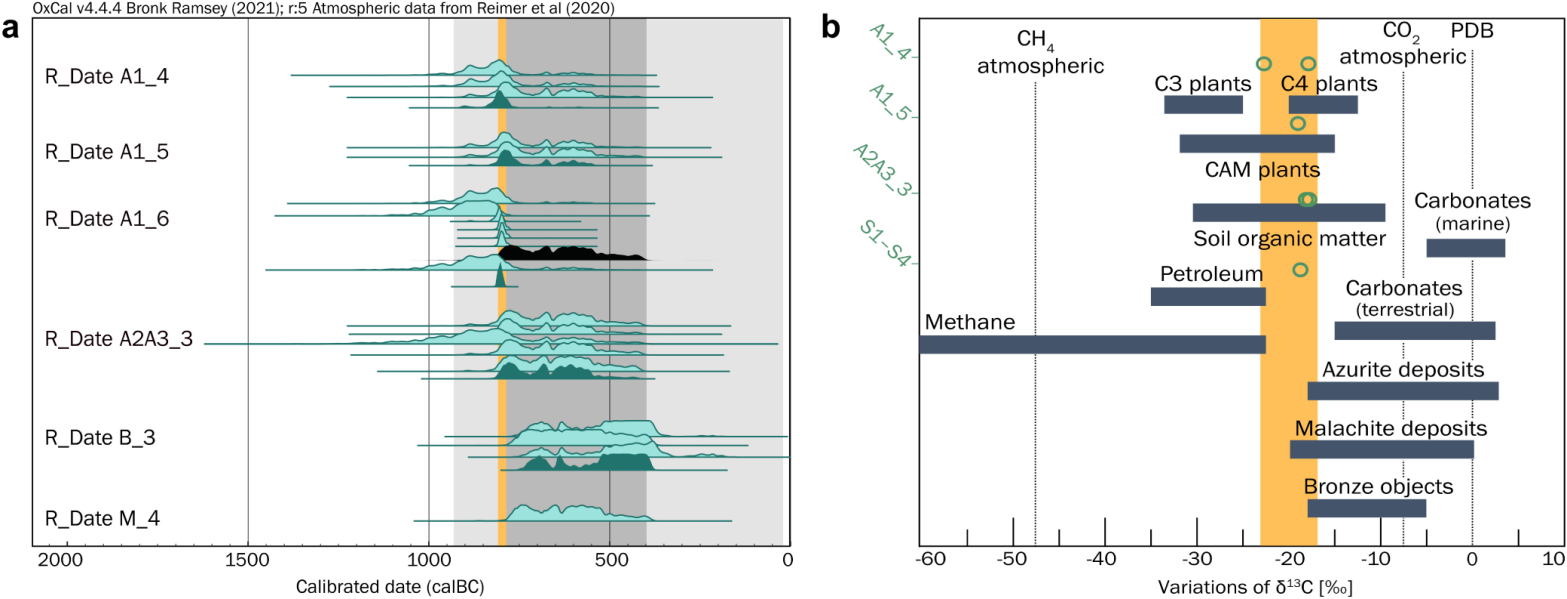
Carbon isotope results. (a) Calibrated radiocarbon ages (95.4% probability range) for the mineralized textile finds from Creney-le-Paradis. For each sample where replicates (light green shade) were measured, averages were calculated (darker green shade), and for sample A1_6, the additional organic residue is shown (black). The orange-shaded area represents the mean value of 2619 ± 11 yr BP, a precise time window covering a period of 18 years. The Iron Age and the estimated period of occupation of the Creney site are shown in light and dark gray, respectively. (b) Comparison of δ^13^C values measured in Creney textile carbonates with characteristic ranges for terrestrial materials. In orange, δ^13^C values of the mineralized textiles (individual measurements are represented by green dots), indicating a spread from −22 to −17‰, typical of plants and soil organic matter. Figure adapted from Trumbore and Druffel^32^ with data from Melchiorre et al.^33–35^

The ^14^C ages obtained on the gaseous fraction extracted from the mineralized carbonates clustered around 2500 radiocarbon years BP, which, when calibrated to real calendar ages, correspond to the Iron Age period. Large uncertainties were induced by the small sample sizes (as little as 25 μg of carbon were measured) and the flatness of the Intcal20 atmospheric calibration curve over this period.^25^ Each individual calibrated result (corresponding to a measurement) spanned a wide period from 800 to 400 BC (95.4% probability range). The lack of contextual archeological data made it impossible to use the position of the material at the site to deduce whether all measurements could correspond to a single event. We therefore tested the statistical combination of the ^14^C ages of individual samples on the basis of the null hypothesis that the dates could not be combined. This showed that all samples could be combined except for sample B_3, which tended to be slightly younger (mean ^14^C age: 2424 ± 41 yr BP, calibrated age range: 752–403 BC; Figure 5a). A1_6 was initially excluded to avoid biasing the results due to its much larger weight (>60 mg) and multiple replicates (*n* = 6, mean ^14^C age: 2629 ± 12 yr BP). The combination of all samples excluding B_3 and the A1_6 replicates indicated a common event that occurred between 807 and 675 BC (R_Combine (2580, 23), χ^2^-test: 10 d.f., *T* = 3.4, 5%: 18.3). The final inclusion of sample A1_6 provided a yet more definite time window, 808–790 BC (R_Combine (2619, 11), χ^2^-test: 19 d.f., *T* = 12.6, 5%: 30.1).

The extremely narrow time window (18 years) provided by the direct dating of the mineralized carbonates raised the intriguing question of the origin of the carbon atoms involved in the growth of the basic copper carbonate crystals. What event were we dating?

### Mineralization Mechanism

Mineralization of the textiles required four steps: (1) the corrosion of a copper-based object as a source of Cu^2+^ ions in the immediate vicinity of the fibers, (2) a source of carbon species (CO_2_ or ${\mathrm{HCO}}_{3}^{-}$ ), (3) the transport of the Cu^2+^ ions and carbon species to the textile, and (4) a mechanism favoring the growth of carbonates preferentially on the surface of textiles (Figure 7).

#### Source of Copper Ions

The copper ions originate from the corrosion of the bronze substrate inside the burial chamber. Transport-induced mineralization confirms that the grave was not watertight. Groundwater infiltration and runoff, or from rising water table, corroded the metal under conditions equivalent to those of a confined indoor atmosphere, possibly subjected to cyclic wet and dry exposure. Analysis of three mineralized textile fragments revealed that the metal substrate was binary copper–tin bronze sheets between 4/10 and 7/10 mm thick, containing significant traces of arsenic and other minor metallic elements (SEM-EDS; SI Appendix Section 2.3). Only very small amounts of calcium and potassium from the soil context were found in the cupric mineralization. In humid indoor atmospheric conditions and shortly after burial, the bronze underwent decuprification, i.e., oxidation of the alloy with preferential dissolution of copper^36,37^ (as confirmed by the relative tin enrichment of the corroded substrate; SEM-EDS). Without leaching by groundwater, but in contact with condensation water, copper cations produced on the metal surface can form a substantial corrosion deposit (up to a few hundred micrometers) after a few years.^38^

#### Source of Carbon

Four distinct origins of carbon could be considered in the formation of the copper­(II) carbonates: (i) the textile fibers themselves, (ii) dissolved inorganic carbon (DIC) derived from the calcareous bedrock of the Champagne crayeuse (“Chalky Champagne”), (iii) atmospheric CO_2_, and (iv) dissolved organic carbon (DOC) or gaseous CO_2_, both derived from the decomposition of soil organic matter or from petrogenic origin.

Dissolved inorganic carbon (DIC) and atmospheric CO_2_ can both be rejected on the basis of incompatible ^14^C and/or δ^13^C signatures (Figure 5). Neither can be considered as the sole contributors, yet the possibility of a minor contamination contribution (below 2%) in the overall carbon source must be acknowledged (as estimated by constant contamination models in Figure S8). The δ^13^C values measured in our copper­(II) carbonates ranged from −22 to −17‰ (Supporting Information Table S3). These values correspond to the typical soil organic carbon δ^13^C signature, between −28 and −10‰ (Figure 5b). Our values are in the lowest range of the δ^13^C values reported for malachite deposits (−21 to 0‰), azurite deposits (−20 to +3‰), and patinas from archeological samples of different origins (−18 to −7‰).^33–35^ In both copper carbonate deposits and patinas of ancient bronze objects, the lowest values were attributed to soil-derived organic compounds, where the classification of the soil and the vegetation present at the site appear to be the dominant factors influencing the δ^13^C values. It is very interesting to note that our values fall not only within these lower values but also within a much narrower range. This is attributed to a closed carbon system—a sealed off environment compared to the diversity of physicochemical mechanisms present in open systems, such as lixiviated soils and corroding objects.

Therefore, two hypotheses remain for the carbon dated in the carbonates: labile carbon present in the near environment—dissolved organic carbon (DOC) or gaseous CO_2_ —from decaying organic matter, or the textile fiber themselves.

#### Transport

Under the environmental conditions during copper ion production, a film or layer of water always covers the metal surface, forming an aqueous electrolyte in which ionic transport took place. Condensation water, and more probably groundwater, played an important role in the transport process. In fact, as soon as surface water, which contains oxygen and free carbon dioxide, came into contact with the bronze sheets, primary oxidation led to the development of solvated cuprous ions which, in solution, rapidly oxidized into stable cupric ions (disproportionation, *K*_*s*_ = [Cu^2+^]/[Cu^+^]^2^ ≈ 10^6^). On the bronze substrate, the result was a local drop in pH to more acidic values and a slight dissolution of the metal.^39^ Under these conditions, as observed here, uniform corrosion of the bronze prevails. This process slows down over time, often stopping after a few years, depending on wet and dry events, because of the formation of a passivation layer. It is even more efficient because of the physical properties of textile fibers, the transport and concentration of ionic species are favored both by capillarity and by evaporation, promoting the formation of a protective corrosion deposit. The exclusive presence of copper­(II) hydroxide carbonates in the mineralization deposit clearly indicates that the dissolved cupric ions were transported from the bronze in an aqueous electrolyte rich in bicarbonate anions, and not in other common copper-complexing agents such as silicate, sulfate, or chloride ions often encountered in archeological contexts.^35,40^ The absence of calcium and silicon-based species in the mineralized fragments reinforces the scenario of rapid transport before the tomb collapsed.

#### Crystallization of Copper Carbonates

In the confined environment and in contact with the wool textile and its high capillarity effect coupled to a high evaporation surface, copper carbonates should become highly enriched in the aqueous phase, exceeding their solubility limit during the evaporation phases, due to the reduction in local water content. Malachite and azurite are highly insoluble in water and precipitate preferentially to copper (hydr)­oxides (CuO, Cu­(OH)_2_, etc.) in carbonate-rich environments.^40,41^ They crystallize under very similar conditions. Their stability depends primarily on p­(CO_2_) and pH. Vink^42^ described azurite as more stable than malachite in relatively acidic (6 < pH < 7) and carbonate-rich (p­(CO_2_) > 10^–3.45^ atm) environments. Malachite appears to be more stable in alkaline media (7 < pH < 8) and carbonate-poor environments (p­(CO_2_) < 10^–3.45^ atm).^42^ As the atmospheric p­(CO_2_) is around 10^–3.4^ atm, slight changes in carbon dioxide partial pressure can easily lead to solid phase transitions from azurite to malachite.^31,41^ Although little is known about the kinetic control of copper carbonate hydroxides, nucleation of malachite and azurite may involve the formation of transient phases such as metastable copper carbonate hydroxide^43^ or the amorphous polymorph to malachite, georgeite (typical composition Cu_2_(CO_3_)­(OH)_2_·6H_2_O).^44,45^

Cupric-ion-laden water-impregnated textile fibers prevent their biodegradation but can also embed them totally or partially. We consistently observed that the shape of the fiber cuticle was preserved (OM, SEM). In some parts of the samples, the textile fibers could be identified individually, which we attribute to the rapid enrichment in cupric ions by sorption, concentration, and precipitation (Figure 6a). In many areas, often closer to the bronze sheet, the fibers are embedded in corrosion products and filled with large, well-crystallized carbonate crystals (Figure 6b). Both facies correspond to a two-stage mechanism in which the fibers were first preserved during the mineralization phase, with carbonate nucleation and growth on the fiber surface, followed by partial degradation and subsequent leaching of the keratin molecules that make up the fibers.

**Figure 6:**
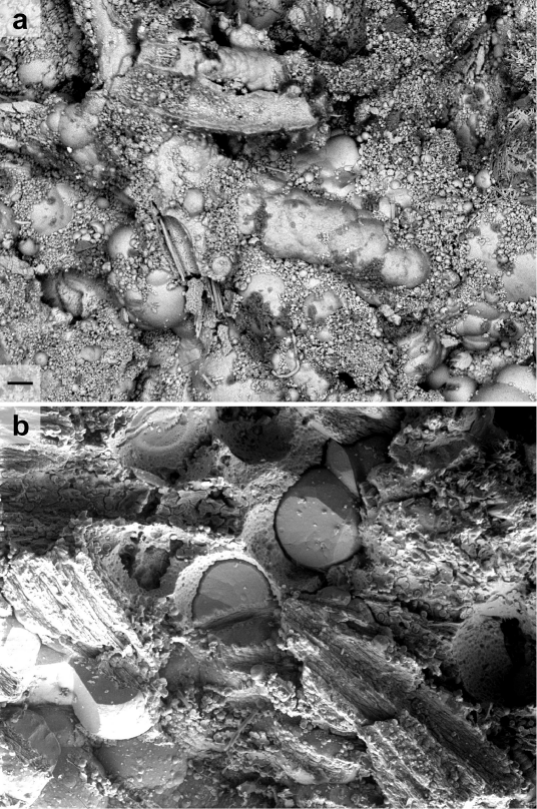
Mineralization facies. Scanning electron (BSE) images of mineralized fibers showing the accumulation of micrometric crystals (sample N7, a), and well-crystallized copper carbonates (sample N12, b); scale bar: 30 μm. In both cases, the cuticle was preserved.

### Association with Dated Event—Adjacent Dating

During nucleation and growth, the tomb environment was “sealed” by the burial context. In fact, the top cover of the tomb, covered with a layer of peaty clay and then with a cap of pieces of chalk^13^ not only prevented any gaseous exchange with the ambient atmosphere, but also limited water infiltration, and therefore any subsequent contamination of the tomb indoor from other carbon sources. In addition, the presence of a plank formwork covering all the walls of the burial chamber must have protected the tomb from the direct chemical decomposition of the limestone (chalk) walls, which is likely to occur in a potentially acidic environment marked by decomposing organic compounds.

Although the amount of carbon involved in carbonates does not, on its own, allow decaying wool textiles to be excluded as a source, our microscopic and taphonomic evidence allows us to reject it. In fact, the alteration of wool proteins is known to proceed through the degradation of amino acid side chains and the hydrolysis of peptide bonds, leading to the production of labile polypeptides.^47,48^ We observed that the original surface of the fibers was well-preserved, with copper hydroxycarbonate hydroxide crystals that nucleated and grew on their cuticle (OM, SEM; Figure 6a). In the case of mineralized wool, the cleavage of peptide bonds by keratinolytic microorganisms and insects is slowed down by the toxicity of the Cu^2+^ ions.^49–51^ In the absence of enzymatic catalysis by microorganisms, it is unlikely that decomposition to the CO_2_ or ${\mathrm{HCO}}_{3}^{-}$ stage would have occurred within the short growth time of the carbonates. Polypeptides must therefore have leached out long after carbonate growth began. We in fact observed a few localized organic pockets of preservation in fiber lumen and in the corrosion layer (SEM; Figure 6). Proteomics proved to be effective in detecting protein fragments in comparable mineralized textiles,^52^ which is in agreement with our observation. These remaining organic compounds were selectively removed using the protocol targeting exclusively carbonate minerals.

The chemical stability of copper carbonate corrosion products (malachite and azurite) is very high. These products are quite insoluble in water at near neutral pH and room temperature, with a very low value of their solubility product constant *K*_sp_ – between −32 and −34 for malachite and around −46 for azurite^53^ corresponding to a solubility of about 3 × 10^–7^ mol L^–1^. Thus, the decomposition–dissolution of basic copper carbonates as well as their reprecipitation involving new basic carbonate species have to be considered as a negligible process here. It should be noted that the conditions for azurite stability will be favored in aqueous environments that are richer in dissolved CO_2_ and more acidic than for malachite.

We therefore conclude that the organic matter in the soil associated with the archeological burial context is at the origin of the measured date, and not the fibers themselves. This had to occur during the short time window (years) during which labile copper ions remained available. Mineral growth ceased when Cu^2+^ ions stopped percolating from the alloy object, either because the corrosion process halted (fully corroded or passivated metal) or (less likely) because ambient humidity decreased due to environmental changes at the site (Figure 7). The limited duration of carbon incorporation and the insolubility of carbonates are the driving force behind the unexpected “date recording” capacity of mineralized organic materials.

**Figure 7:**
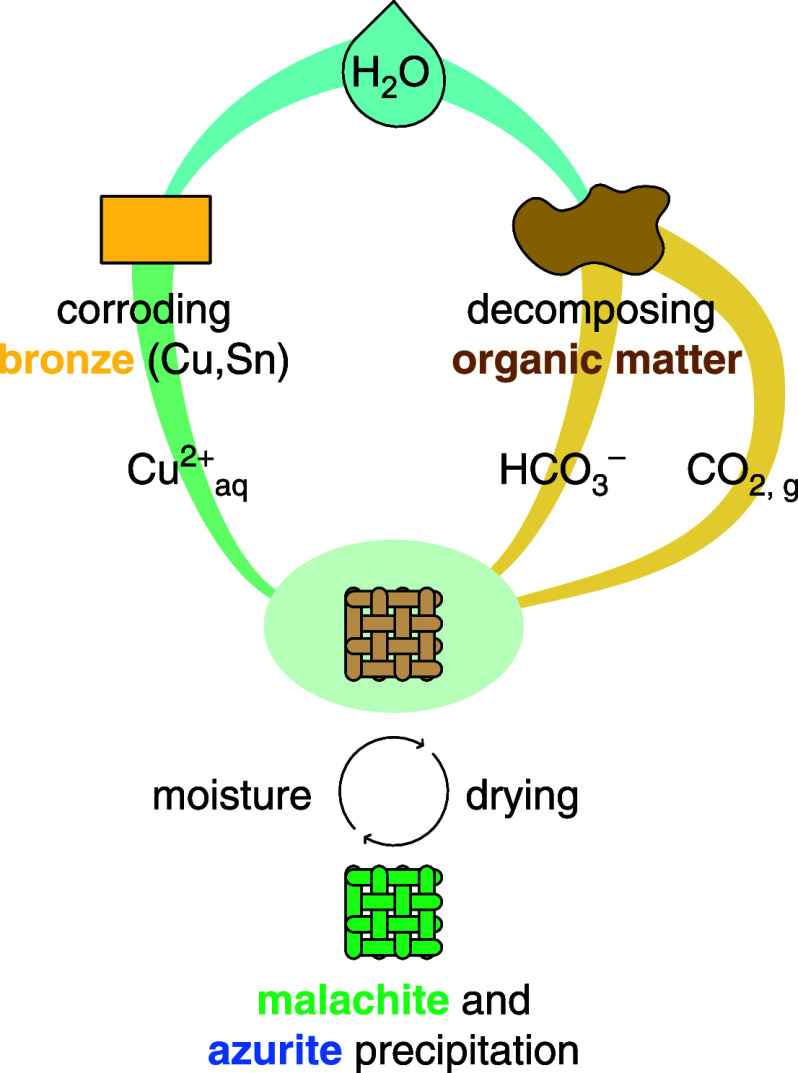
Schematic representation of the mechanism leading to fiber mineralization. The water could have come from percolation through the structure, runoff from the walls, rising groundwater, flooding from neighboring marshes, or condensation.

Our ^14^C and δ^13^C measurements reflect the isotopic composition of the burial environment; which are human-made. The stored ^14^C age in the carbonate accretions corresponds to the moment when the biomass consumed to produce labile carbonates ceased to interact with the global carbon cycle. Our interpretation therefore points toward a main scenario: the carbonaceous material arose from a short time sequence when many plants were isolated from the global carbon cycle. The site is very close to the Villechétif locality (also known as Ville-Chétif), where is located the Argentolle marsh,^54^ a local peat bog documented since at least the 1870s for its prehistoric settlements^55^ (Figure 8).

**Figure 8:**
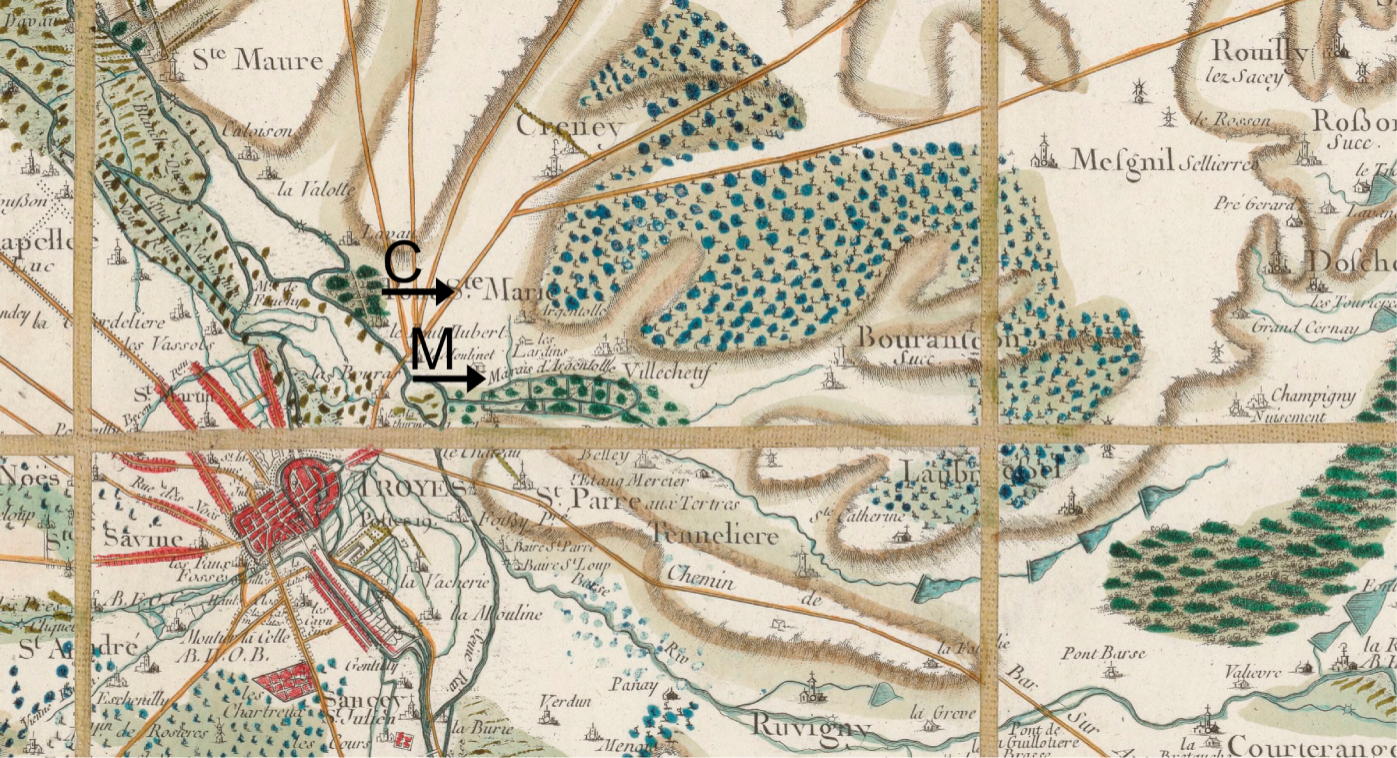
Close-up of the Cassini map dated 1758–1760. The Creney-le-Paradis site (marked C) is located to the northeast of Troyes, around 1 km from the Argentolle marsh (M). Adaptation of the *Carte générale de la France* edited by César-François Cassini de Thury and engraved by Louis-René Luce, N^◦^81. Flle 31e, Bibliothèque nationale de France, ark:/12148/cb40860575c.

Interestingly, archeologists noted that the third burial mound (i.e., third and last phase of construction of the tumulus) was covered with “tiles” of brown organic compounds (peat) and a layer of chalky sediment.^13^ The infill material of the burial chamber consisted of “compact dark brown to reddish clay, mixed with loose dark brown fine earth” of the same nature as these tiles.^13^ Indeed, the archeologists noted that “the construction of the first central mound, 8 m in diameter, consists of a succession of layers of chalk-rich soil and very organic, fine, dark brown soil from the marsh.”. We therefore assume that we are dating the carbon from this humus-rich decomposition soil, dated to the eighth century BC, fortuitously captured in the mineralization of the textiles.

While no previous study had explored the potential of copper corrosion products as potential time capsules, this development reminds us that beyond traditional carbon-rich materials (wood, charcoal, textiles, bone), ^14^C dating can trace carbon in the carbon cycle over a much wider range of substrates. For example, calcium carbonate, whether from foraminifera or mortars, is known to preserve chronological information in their carbonate anion.^1^ The most widely used historical pigment in the arts, lead white, a carbonate pigment (2 PbCO_3_ · Pb­(OH)_2_), was also shown to retain the signature of atmospheric CO_2_ during its production.^56–58^ The formation of the humus-rich soil (or peat) precedes its use, the proposed date of 808–790 BC is therefore a *terminus post quem* in archeological terms. This date supports current stylistic evidence indicative of the first foundation of the tumulus in the eighth c. BC.^13^ The date found likely corresponds to the organic materials used for the floor of the first burial mound. The use of burial structures over relatively long periods of time is known in contemporary contexts, particularly through the installation of chronologically staggered tombs in the same burial mound (e.g., La Ronce tumulus).^59^ This likely implies an intention to integrate the dead in a perceived continuity, a collective memory real or imagined, organized by his successors for dynastic reasons.^60^ We cannot completely rule out the alternative scenario whereby material dating from 808 BC was incorporated when the central pit was constructed in the mid sixth to fifth century BC. In any case, the results obtained here open up the fascinating prospect of being able to specify the chronology in which genealogical or relational (re)­construction between individuals took place and to better understand the series of sequences and gestures that enacted this intention.

## Conclusions

We propose to use carbon-14 dating in an unconventional way, by studying analytes whose temporal sequence of carbon integration can be reliably interpreted. Carbonates from mineralized organics can act as time capsules. In the case of Creney, we argue that our method provides highly accurate dates associated with events that occurred during the foundation of the site. The chronological information could not be obtained using traditional radiocarbon protocols, which only target organic matter and required a tailored thermal preparation. Reliable ages were obtained, which means that the two essential criteria for radiocarbon dating were met: (i) the carbon measured was at some point in stationary state with atmospheric CO_2_ and, (ii) the mineralization mechanism behaved like a closed system—after formation of carbonate corrosion products, no secondary  carbon was added nor exchanged. The mineralizing textiles acted as passive samplers, which now allows us to suggest a narrow time interval for the organic matter deposited when the mound was first founded. While our results show internal consistency and align with archeological data, comparison with independently dated materials will be essential to validate this approach across different contexts.

We believe that this study could stimulate the revision of many archeological cases in order to gather more complete evidence on the origin and development of sites, following the example of the movement observed in cold case forensics with new DNA microanalysis procedures. Many mineralized organic materials result from a sudden “catastrophic” taphonomic event, which could be approached as potential time capsules. Although our work focused on mineralized textiles, the adaptation of CO_2_ extraction from tiny samples of copper carbonates could be extended to the study of other mineralized matrices such as corrosion crusts. We argue that microscopic analysis could be used, not only to provide direct dating of artifacts but also to map complex in situ processes in archeology.

## Supplementary Material



## Data Availability

The XRD, TGA and ^14^C dating data supporting the conclusions of this study are openly available on Zenodo at 10.5281/zenodo.17817805. The Oxcal software is available at https://c14.arch.ox.ac.uk/oxcal.html.

## Notes

Additional data on the archeological site, a detailed description of the materials, detailed XRD and SEM-EDS data, and detailed results of radiocarbon and stable isotope analyses (PDF)
