# Supplementary material for: Mineralized Remains as Adjacent Proxy for Radiocarbon Dating
Source: Anal Chem. 2026 Jan 14;98(3):1880–90. doi: 10.1021/acs.analchem.5c03812 (PMC12856835; doi:10.1021/acs.analchem.5c03812)
Supplement: Supplementary file 1 [file ac5c03812_si_001.pdf]

Supporting Information:

Mineralised remains as adjacent proxy for radiocarbon dating

Laura Hendriks,[*,†] Clémence Iacconi,[†,‡] Luc Robbiola,[¶] Elsa Desplanques,[§] Negar Haghipour,[‖,⊥] Corentin Reynaud,[‡] and Loïc Bertrand[*,‡]

†*HEIA School of Engineering and Architecture of Fribourg, HES-SO University of Applied Sciences and Arts Western Switzerland, Pérolles 80, 1700 Fribourg, Switzerland*

‡*Université Paris-Saclay, ENS Paris-Saclay, CNRS, PPSM, 4 avenue des sciences, 91190 Gif-sur-Yvette, France*

¶*TRACES, CNRS, ministère de la Culture, Université Toulouse–Jean Jaurès, UMR 5608, 31000 Toulouse, France*

§*Sorbonne Université, Centre André Chastel, 2 rue Vivienne, 75002 Paris, France*

‖*Laboratory of Ion Beam Physics, ETH Zurich, Otto-Stern-Weg 5, 8093 Zurich, Switzerland*

⊥*Geological Institute, ETH Zurich, Sonnegstrasse 5, 8092 Zurich, Switzerland*

E-mail: laura.hendriks@hefr.ch; loic.bertrand@ens-paris-saclay.fr

Contents

1 **Supplementary information on the Creney-le-Paradis site** **S-3**

2 **Detailed material description** **S-3**

2.1 Textile fragments S-3

2.2 Mineralization facies of mineralized textile fragments S-3

2.3 Composition of the metal substrate S-9

3 **Radiocarbon dating** **S-9**

4 **Stable carbon isotope analysis** **S-14**

References **S-16**

1 Supplementary information on the Creney-le-Paradis site

The archaeological site at the Le Paradis locality of Creney-près-Troyes (Creney-le-Paradis, Aube, France) contains two large groups of archaeological structures: (i) a necropolis used from the Final Bronze Age to the beginning of the late Iron Age (c. 8th–5th BC), and (ii) an indigenous farm associated to a Gallo-Roman occupation[S1]. Excavations at the necropolis revealed several phases of concentrically superimposed construction of a tumulus with a large central burial chamber (2.1 m × 3 m) with a formwork of vertical planks set into the chalky soil.

2 Detailed material description

2.1 Textile fragments

The 99 fragments of mineralized textiles identified in the material from the central pit of Creney-le-Paradis range between 6 mm × 3 mm (smallest) to 44 mm × 18 mm (largest). The mineralized textile remains are wool and show color variations from green to light or dark blue (Fig. S2). 54% showed predominantly blue corrosion products, 23% green corrosion products and 23% a mixture of both. X-ray diffraction analysis identified the main mineral phases as malachite $Cu_2(OH)_2CO_3$ and azurite $Cu_3(OH)_2(CO_3)_2$. Three different types of weave were identified throughout the entire corpus: twill (87% of the determined textiles), tablet-weaving (12%) and tabby (one occurrence)[S2]. Because of their flat shape, these remains were associated with a bronze object shaped by hammering. They were interpreted as remains from metal-urn cremation burial[S2].

2.2 Mineralization facies of mineralized textile fragments

The observation of mineralized textile fragments was carried out without any surface preparation, particularly on three representative fragments (N7, N8, N12). As shown in Figure S4, deposits of copper corrosion products can vary in thickness but always reveal the presence of malachite (green, $Cu_2(OH)_2CO_3$) and azurite (blue, $Cu_3(OH)_2(CO_3)_2$). Pseudomorphic fibers are often observed in

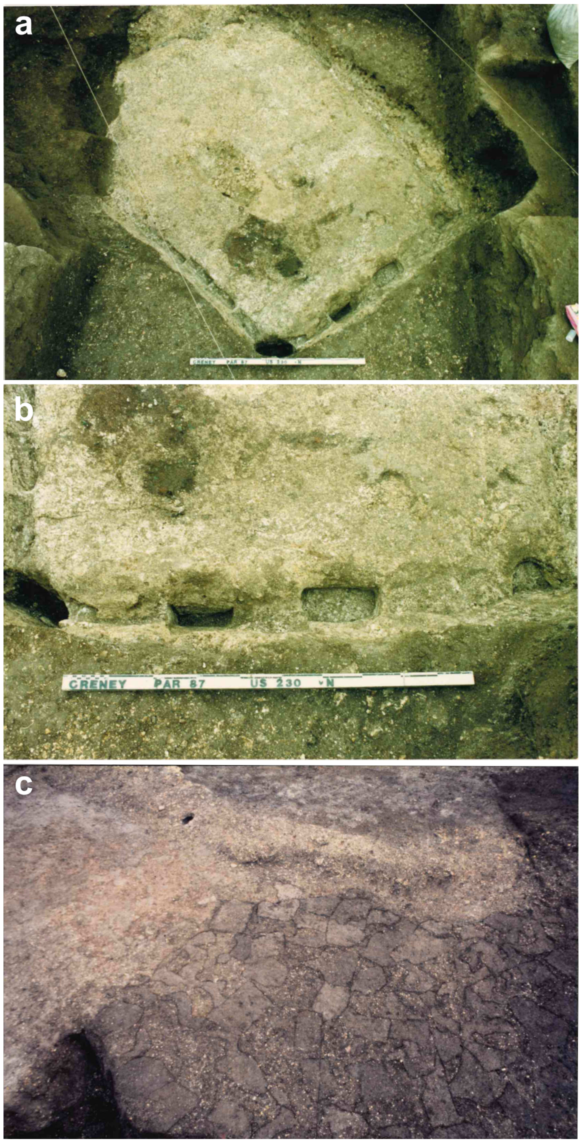

Figure S1: **The site of Creney-le-Paradis (Aube, France).** (a) Burial, central pit. Overview of the excavated burial chamber; scale bar: 1 m. (b) Detailed view of the edge of the chamber, showing the imprints of the wooden formwork; scale bar: 1 m. (c) Plan view of the ‘tiles’ of fine, dark earth alternating with a chalky sediment forming the third mound. On the right: construction in dark earth; left: in chalky earth. Figures adapted from Villard et al.[S1].

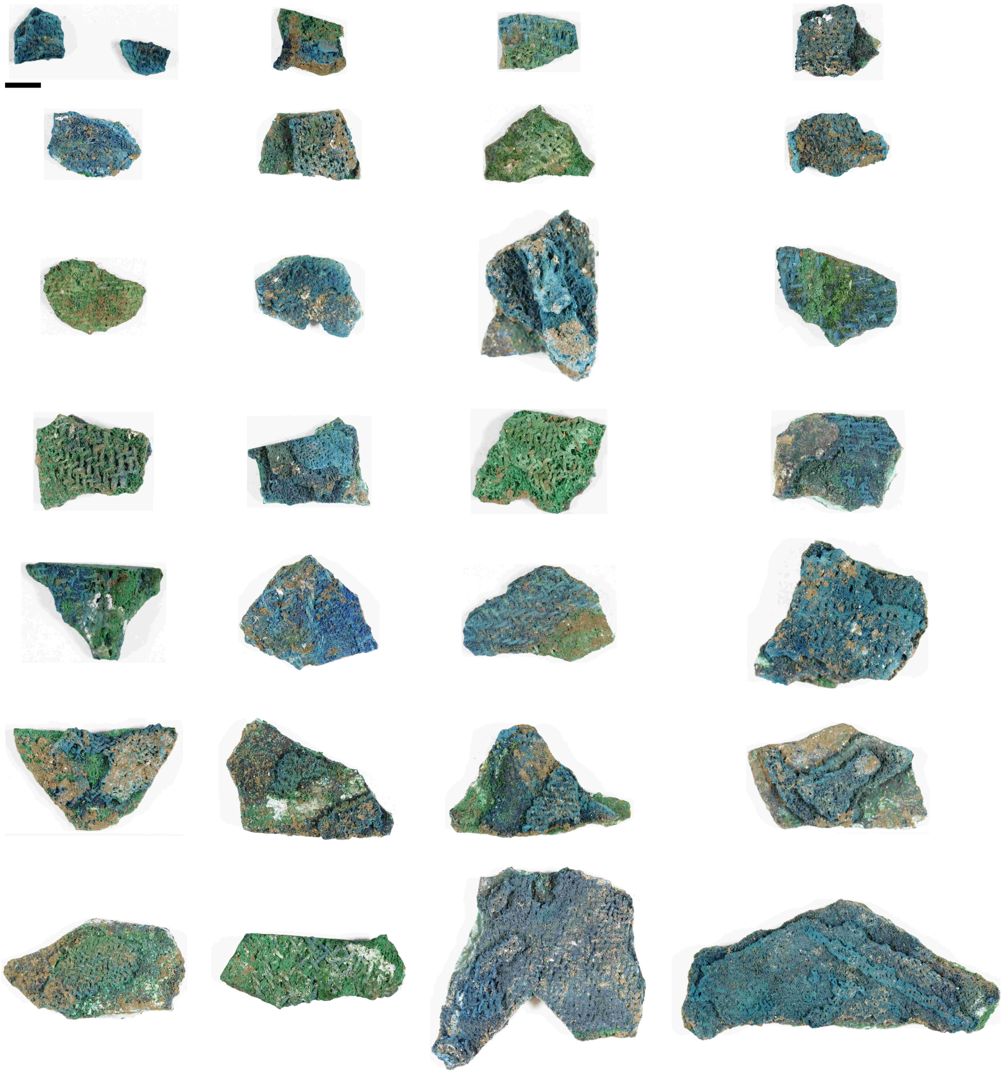

Figure S2: **Mineralized textile fragments.** Macrophotographs of 29 out of the 99 fragments of mineralized fragments found at the Creney-le-Paradis site (Aube, France). Scale bar: 5 mm.

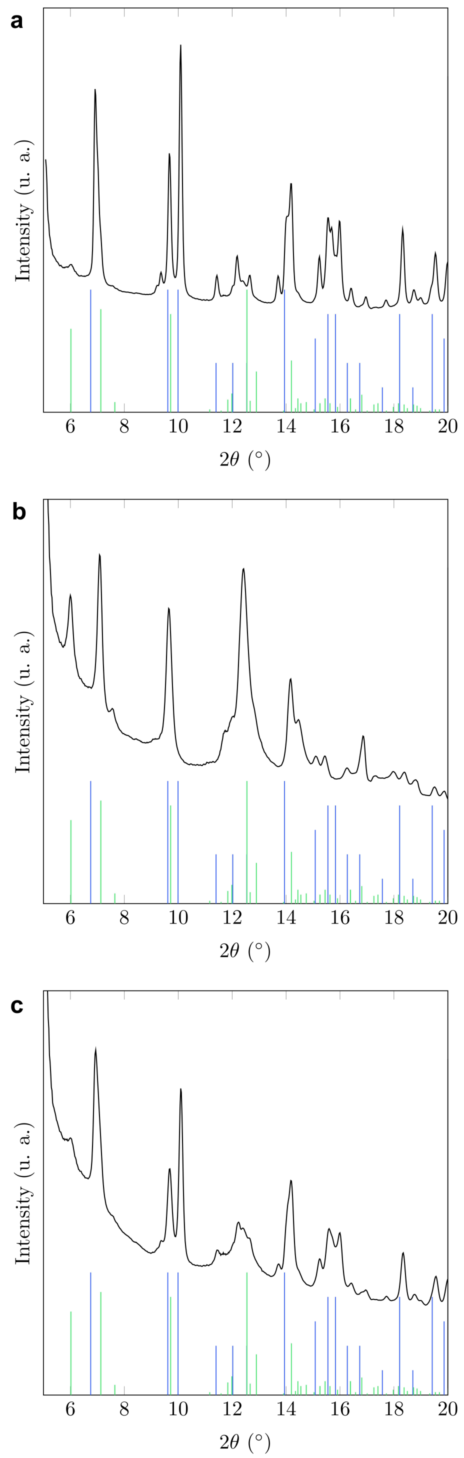

Figure S3: **Synchrotron-based diffraction patterns collected on mineralized fibers from Creney-le-Paradis (Aube, France).** X-ray diffraction diagrams of three textile fiber samples: A1_4 showing mainly azurite (a), A1_5 showing mainly malachite (b), A2A3_3 showing a mixture of both (c). All samples show the combined presence of the two mineral phases, regardless of their predominant mineral color. The green bars represent the reference diffraction pattern for malachite, while the blue bars represent those for azurite.

azurite, resting on green copper products on the surface of the corroded sheet (Fig. S4a,c). Mineralized fibers are often difficult to identify in detail. BSE images revealed very little contrast between the surface corrosion deposits with or without fibres, as shown in Figure S4b,d corresponding to the areas in Figure S4a,c respectively.

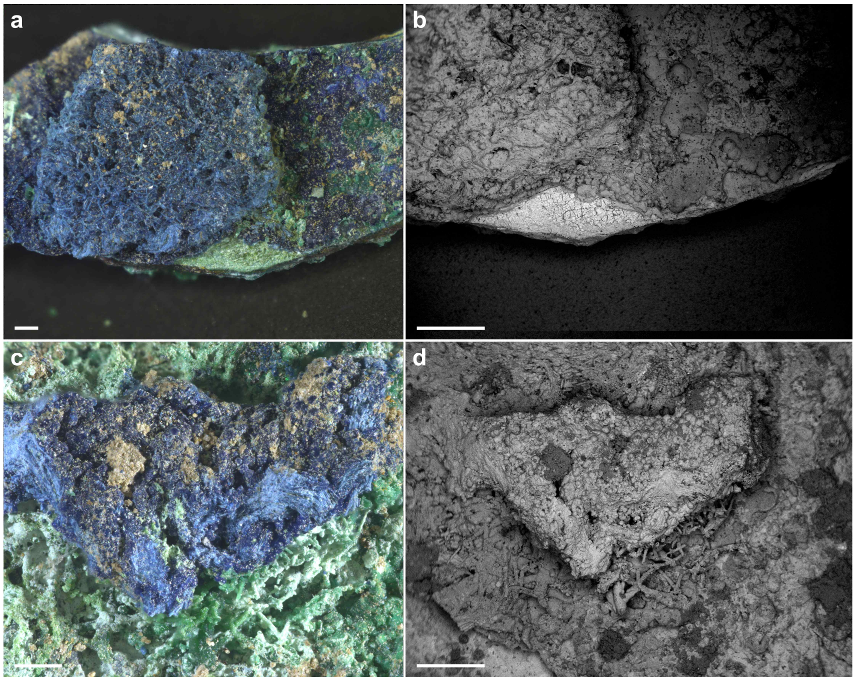

Figure S4: **Observation of the surface of copper corrosion deposits with mineralized fibers.** Optical microscope images of mineralized textile fragments N12 (a) and N7 (c); all scale bars: 500 µm. BSE images of mineralized textile fragments N12 (b) and N7 (d). Green malachite and blue azurite crystals associated with mineralized fibers appear in gray, corroded bronze in white and residual soil minerals in dark gray. All scale bars: 1 mm.

Several features were observed that confirm the rapid growth and formation of copper(II) species deposits on the corroded bronze sheet. As shown in Figure S5, detailed study of the cross-sections of fragments N7 and N12 shows that the copper(II) deposit is very homogeneous in composition but irregular in shape, with numerous porosities.

This is also observed between the deposit and the corroded bronze substrate. This indicates that a rapid growth rate led to the embedding of textile fibres, rather than a slow growth rate which would have led to a thick, even layer of corrosion, as is usually observed in ancient bronze patinas[S3,S4].

In some cases, radial aggregates of botryoidal shape forming block aggregates are revealed

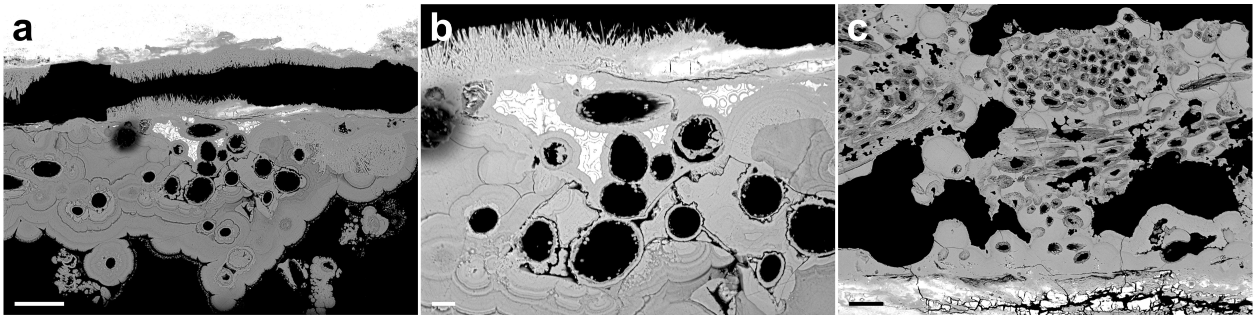

Figure S5: **Observation of the internal morphology of copper(II) hydroxycarbonate deposits on cross-sections, with BSE electron microscopy.** (a) Mineralized textile fragment N7. (b) Detailed view of the central zone of image (a). (c) Mineralized textile fragment N12. SEM imaging reveals coalesced crystals (gray), surrounding ghost textile fibers and large internal porosities (black), located between these elements and the corroded bronze sheet (white). In addition, secondary crystallization, such as acicular crystals, can be observed within these porosities and on the primary deposit. Scale bars: 100 μm except in (b): 20 μm.

(Fig. S6a), which may be linked to a nucleation and growth process that takes place in a single step. There is also evidence that on these primary formations of copper(II) carbonate species, secondary precipitation can sometimes be observed in the porosities of the primary deposit, as shown in Figure S6, such as curly malachite (Fig. S6b) typically observed in a confined humid atmospheric archaeological environment[S5] or tabular azurite Fig. S6c).

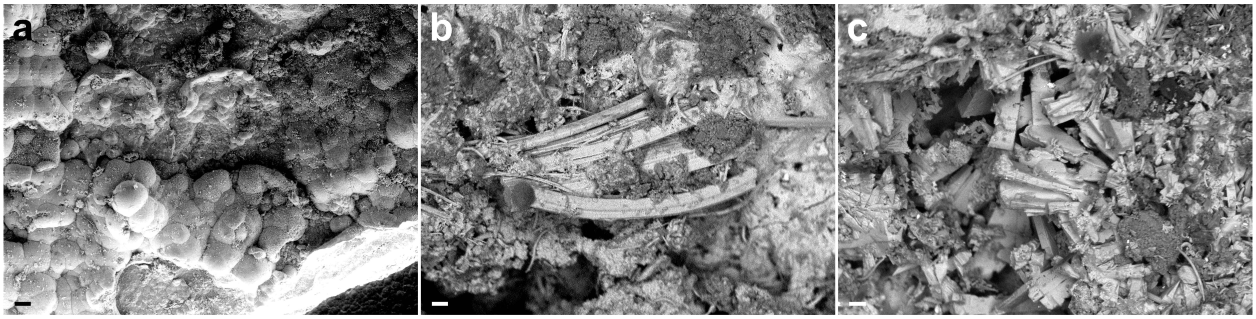

Figure S6: **Copper(II) deposit morphologies on the surface of bronze sheets surrounding mineralized fibres.** BSE images of radial botryoidal malachite aggregates (N12) (a); curly malachite fibers (N8) (b), and radiating clusters of tabular to prismatic platelet-shaped azurite crystals on a blocky malachite matrix (N8) (c). Scale bars: 20 μm except in (a): 100 μm.

2.3 Composition of the metal substrate

Observation of the sections of the fragments revealed that the green and blue copper deposits are irregular in thickness and sometimes porous (Fig. S7). The limit of the original surface is still clearly visible. It is marked by direct contact with a thin green copper deposit on the outer part and corrosion layers on the inner part. This morphology, which preserves the original shape of the metal sheet, is related to a uniform corrosion process that is often observed in bronze alloys[S3]. Elemental analysis of the corroded sections confirmed the tin bronze nature of all these metal sheets. The Cu/Sn weight ratio measured in the central part of all these bronzes ranged from 1.5 for the most corroded (N7) to 7.9 for the least corroded (N12). Sheet thickness was measured at around 400–450 μm for N8 and around 480–520 μm for N7 and N12. In addition, semi-quantitative elemental analyses of the three mineralized textile fragments identified the metal substrate as pure binary bronze (Cu–Sn alloy) and showed the presence of As as significant trace elements (As/Sn weight ratio between 0.01 and 0.03). A few other possible trace elements, Ni, Ag and Pb, remain below the limit of quantification (< 0.1 wt%).

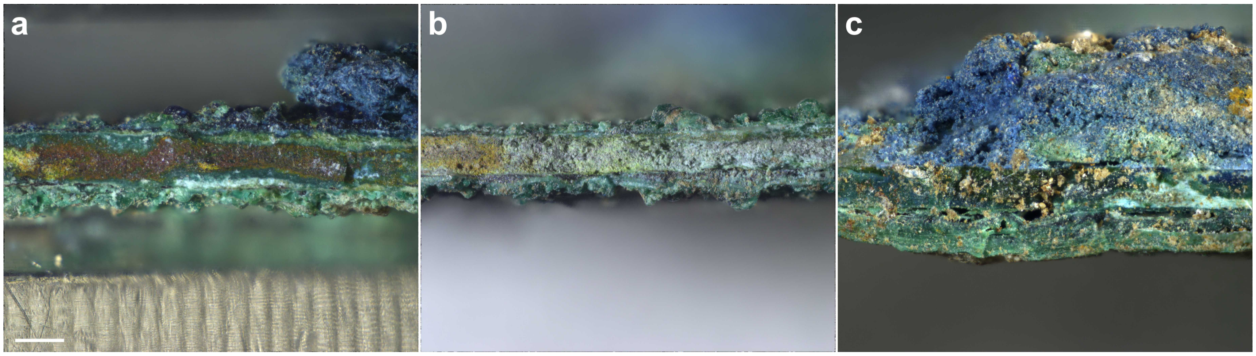

Figure S7: **Section of mineralized textile fragments.** Optical microscope image of the section of mineralized textile fragment N12 (a), N7 (b) and N8 (c). Scale bar: 500 μm.

3 Radiocarbon dating

Copper(II) hydroxide carbonates consist of more than 50wt.% copper and less than 10wt.% carbon, specifically malachite and azurite contain only 5 to 7wt.% C each. Coupled with microscopic

observations, whether considering a fully or partially mineralized sample and the mineral phase in presence, a theoretical carbon content was calculated from the initial sample's weight (Table S1). For example, samples A1_4 and A2A3_3 show a predominance of azurite, thus the maximal theoretical carbon content is 7% of the total sample mass of the sample. A partial mineralization was observed for sample A1_4 which is reflected in the lower carbon content recovered. In contrast, a 97% yield was obtained for sample A2A3_3, which displayed a negative mineralization state. For the same sample, no carbon was evolved in the EA-AMS, indicating a lack of organic matter and hence confirming the observed mineralization state. The carbon recovery was found to be proportional to the sample mass and in agreement with the mineralization state reported.

Table S1: Efficiency of the selective thermal dissociation approach.

		Mineral			Carbon mass [mg]		
**Sample**	**Mineral phase**	**% C**	**State of mineralization**	**mass [mg]**	**theoretical**	**obtained**	**% recovery**
**A1_4**	azurite	7	partial	7.100	0.497	0.423	85
**A1_5**	malachite	5	positive	1.853	0.093	0.128	138
**A1_6**	malachite	5	partial	65.00	3.250	2.990	92
	azurite	7	partial	0.654	0.046	0.038	84
**A2A3_3**	malachite	5	negative	5.847	0.293	0.265	91
	azurite	7	negative	1.179	0.083	0.080	97
**B_3**	azurite	7	positive	3.795	0.266	0.265	75
**M_4**	azurite	7	negative	0.614	0.043	0.039	91

A total of six fiber samples were analyzed (Table 3); samples A1_6 and A2A3_3 were further split into two subsamples according to the mineral phase observed (malachite vs azurite). Depending on sample mass, multiple preparations were conducted. The samples weighed between less than 1 mg up to over 60 mg for the largest (A1_6), yielding between 0.025 mg to more than 3 mg of C upon thermal dissociation of the carbonates. Based on a similar sample preparation of basic lead white, no isotopic fractionation was expected in the thermal sample preparation[S6,S7], the conservation of the biomass $\delta^{13}C$ values within the carbonate anion further attests to the reliability of the ^{14}C measurement.

The precision of the AMS analysis depends on the counting statistics, typically samples bearing more than 200 µg C, which are measured as graphite can be measured down to a precision of

2‰[S8], while measurement uncertainties for smaller samples range from 0.5–2%. Due to the relatively large uncertainties associated with the small sample size and the plateau like feature of the calibration curve, the calibrated results of the individual measurements span from 800 to 400 BC.

Whether all measurements belong to the same event or not is unclear, due to the lack of information regarding the samples recovered at Creney. A null hypothesis test was conducted using the `R-combine` function in `Oxcal`, where a pooled mean χ^2 test is calculated[S9], a useful tool when comparing ^{14}C dates from archaeological sites[S10]. The mean value of all ^{14}C dates fails the χ^2-test, thus indicating that one sample or more does not originate from the same time period. Visually, sample B_3 does not group with the other set of samples (mean ^{14}C age = 2424 $\pm$ 41 yr BP, calibrated age range: 752-403 BC), an observation which was further confirmed by the null hypothesis test. While sample A1_6 fits the other dates, it also biases the statistic, being extremely larger. Hence, the mean/pooled value of samples A1_4, A1_5, A2A3_3, M_4 indicates a common event that occurred between 807–760 BC (R_Combine (2581,23), χ^2-Test: df = 10 T = 3.4 (5% 18.3)).

In the present work, we propose that the ^{14}C measurements of the carbonate fraction of mineralized textiles can provide absolute age constraints in archaeological sites in the absence of organic matter. In the establishment of this new proxy, it is essential to address the reliability of the resulting ages, specifically considering the source of carbon and possible contamination. The occurrence of geologic and/or secondary carbonate ($0 < F^{14}C < 1$) could potentially compromise the integrity of this approach.

The consistency of the measured data, particularly the absence of bias in the obtained ages, constitutes the first line of evidence supporting a single carbon source. This consistency suggests that the samples were not affected by significant contamination. The relatively narrow spread of the ^{14}C age distribution further strengthens this argument, as any contamination would introduce greater variability in the results. In the presence of various incoming carbon sources, one would expect a broader, more erratic distribution. Additional evidence against potential contamination is provided by the constant contamination models presented in Fig. S8. These models simulate various contamination scenarios and demonstrate that extreme contamination (e.g. geological contamination

with $F^{14}C$ values ranging from 0.005 to 0.1) would have a significant impact on the derived ages beyond 2% threshold. Such contamination would lead to older apparent ages due to the presence of "dead" carbon. Similarly, any contamination from modern environmental carbon sources, as modeled with $F^{14}C = 1$, would result in younger apparent ages beyond a 1.5% threshold. The statistical consistency of the data and substantial absence of age bias, refutes any form of contamination above 2%.

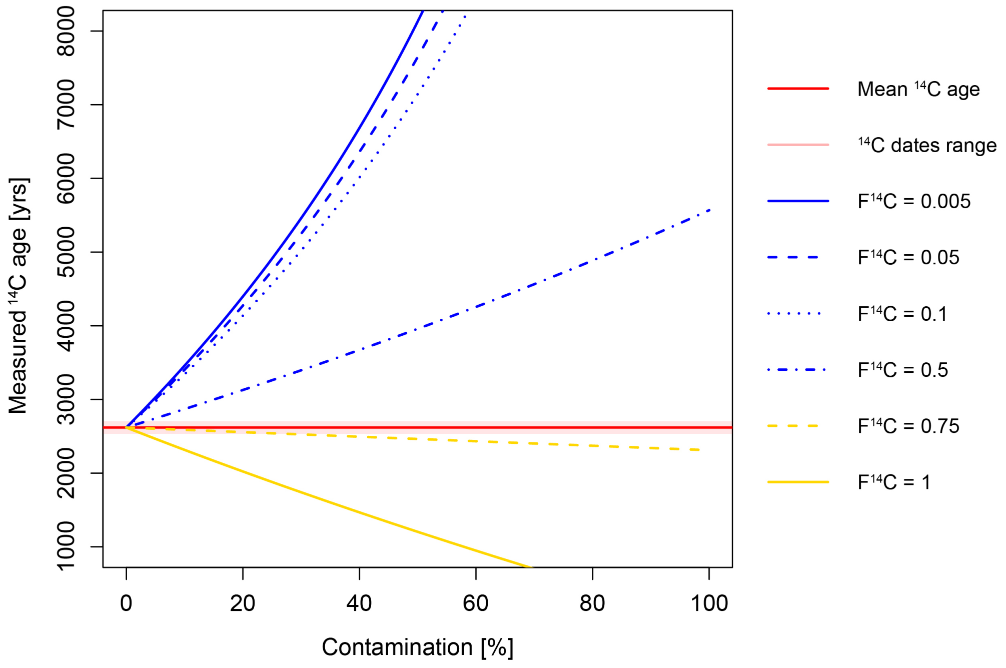

Figure S8: **Impact of contamination on measured radiocarbon ages.** The red area represents the spread of ^{14}C ages, while the red horizontal line highlights the mean ^{14}C aged obtained for our study (2619 ± 11 yr BP, n = 20). The dashed lines represent different constant contamination model scenarios with geologic ($F^{14}C = 0.005$–0.1) and /or secondary carbonate occurring by environmental carbon ($F^{14}C = 0.1$–1).

If $F^{14}C = 0.005$ / ca. 40'000 yrs old, 0.5% contamination goes undetected with 40 years uncertainty, up to 2% if looking at higher boundary given by uncertainty of oldest measured sample. If $F^{14}C = 0.05$ / ca. 25'000 yrs old, 0.6% contamination goes undetected with 40 years uncertainty, up to 2% if looking at higher boundary given by uncertainty of oldest measured sample. If $F^{14}C = 0.5$ / ca. 5500 yrs old, 2% contamination goes undetected with 40 years uncertainty, up to 7%

if looking at higher boundary given by uncertainty of oldest measured sample. When considering a modern contamination, if $F^{14}C = 1$ (modern carbon source), 1.5% contamination goes undetected with 40 years uncertainty. The consistency of the measured data attests to the absence of DIC contamination through hydrological processes, which, if present, would have led to inconsistent or erratic results. The observed stability in the age distributions supports the interpretation that the samples remained free from such external contamination.

Table S2: **Radiocarbon dating results**. The collected data is organized by laboratory code, recovered carbon mass, ^{14}C ages with 1σ uncertainty and fraction modern ($F^{14}C$). Samples were calibrated using Intcal20 atmospheric calibration curve[S11].

Sample	ETH label	C mass (μg)	$\delta^{13}C$ AMS (‰)	^{14}C age ± 1σ (years BP)	$F^{14}C \pm 1\sigma$	Calibrated age range (95.4% CI)
**A1_4**, Azurite (7.1 mg)	131938.1.1	170	-14.6	2647 ± 72	0.719 ± 0.006	989–548 BC
	131938.1.2	120	-14.7	2586 ± 70	0.725 ± 0.006	901–485 BC
	131938.1.3	100	-14.1	2631 ± 70	0.721 ± 0.006	971–544 BC
	**Mean** (χ^2-test: 2 d.f., $T = 0.4$, 5%: 6.0)			**2621 ± 41**	**0.721 ± 0.004**	**899–596 BC**
Organic remain	132042.1.1	2	–	–	–	–
**A1_5**, Malachite (1.85 mg)	131939.1.1	68	-16.5	2596 ± 69	0.724 ± 0.006	908–516 BC
	131939.1.2	60	-17.9	2578 ± 72	0.726 ± 0.006	899–425 BC
	**Mean** (χ^2-test: 1 d.f., $T = 0.0$, 5%: 3.8)			**2587 ± 50**	**0.725 ± 0.005**	**891–544 BC**
Organic remain	132043.1.1	1	–	–	–	–
**A1_6**, azurite (0.65 mg)	131940.1.1[a]	38	-14.6	2682 ± 89	0.7162 ± 0.008	1107–547 BC
**A1_6**, Malachite (65 mg)	131941.1.1	135	-13.1	2671 ± 72	0.717 ± 0.006	1012–572 BC
	131941.1.3	60	-13.6	2727 ± 71	0.712 ± 0.006	1050–786 BC
	131941.2.1[b]	400	-18.1	2645 ± 23	0.719 ± 0.002	891–788 BC
	131941.3.1[b]	776	-14.2	2619 ± 23	0.722 ± 0.002	814–777 BC
	131941.4.1[b]	825	-14.4	2615 ± 23	0.722 ± 0.002	812–777 BC
	131941.4.2[b]	717	-12.7	2619 ± 24	0.722 ± 0.002	816–776 BC
	**Mean** (χ^2-test: 5 d.f., $T = 3.5$, 5%: 11.1)			**2629 ± 12**	**0.719 ± 0.001**	**811–792 BC**
Organic remain	132044.1.1[c]	48	–	2545 ± 86	0.728 ± 0.008	795–412 BC

Continued on next page

Table S2: **Radiocarbon dating results**. The collected data is organized by laboratory code, recovered carbon mass, ^{14}C ages with 1σ uncertainty and fraction modern ($F^{14}C$). Samples were calibrated using Intcal20 atmospheric calibration curve[S11]. (Continued)

Sample	ETH label	C mass (μg)	δ^{13}C AMS (‰)	^{14}C age ± 1σ (years BP)	$F^{14}C \pm 1\sigma$	Calibrated age range (95.4% CI)
**A2A3_3**, Malachite (5.85 mg)	131943.1.1	110	-15.6	2554 ± 76	0.728 ± 0.007	828–416 BC
	131943.1.2	90	-16	2571 ± 71	0.726 ± 0.006	895–421 BC
	131943.1.3[c]	25	-19.6	2665 ± 118	0.718 ± 0.011	1119–422 BC
	**Mean** (χ^2-test: 2 d.f., $T = 0.7$, 5%: 6.0)			**2580 ± 48**	**0.724 ± 0.005**	**828–544 BC**
Organic remain	132046.1.1[d]	24	–	-		
**A2A3_3**, Azurite (1.18 mg)	131942.1.1	40	-16.9	2552 ± 74	0.728 ± 0.007	824–417 BC
	131942.1.2	40	-18.1	2529 ± 73	0.73 ± 0.007	807–418 BC
	**Mean** (χ^2-test: 1 d.f., $T = 0.0$, 5%: 3.8)			**2540 ± 52**	**0.729 ± 0.005**	**808–486 BC**
Organic remain	132045.1.1	0	–	–	–	–
**B_3**, Azurite (3.8 mg)	131944.1.1	84	-17.4	2415 ± 71	0.74 ± 0.007	765–393 BC
	131944.1.2	75	-16.3	2484 ± 70	0.734 ± 0.006	780–413 BC
	131944.1.3	50	-19.1	2373 ± 70	0.744 ± 0.006	762–233 BC
	**Mean** (χ^2-test: 2 d.f., $T = 1.3$, 5%: 6.0)			**2424 ± 41**	**0.739 ± 0.004**	**752–401 BC**
Organic remain	132047.1.1	1	–	–	–	–
**M_4**, Azurite (0.61 mg)	131945.1.1	39	-15	2525 ± 66	0.73 ± 0.006	804–421 BC
Organic remain	132048.1.1	4	–	–	–	–

[a] Carbonate sample (< 40 μg C) was corrected following the model of constant contamination using the following correction parameters $F^{14}C_C = 0.37 \pm 0.05$ and $m_C = 1.6 \pm 0.4$ μg C.

[b] Samples (> 200 μg C) were measured as graphite targets.

[c] Organic fraction remain measured on EA-AMS and corrected following model of constant contamination using the following correction parameters $F^{14}C_C = 0.50 \pm 0.08$ and $m_C = 0.8 \pm 0.2$ μg C.

[d] Sample considered as lost, as the vial containing the cleaned sample fell and broke, which when analyzed showed a modern contamination (dust) hence not included in the table.

4 Stable carbon isotope analysis

Although AMS analysis typically monitors all three carbon isotopes, where ^{13}C is used as a correction parameter for ^{14}C fractionation, the overall design of AMS sources does not allow the measurement of ^{13}C with sufficient precision to ascribe the source of carbon-based molecules.

Hence, complementary analyses were conducted. The $\delta^{13}C$ value of a sample reflects the isotopic composition of the immediate environment and therefore is a crucial indicator as to the source of the sample[S12]. Atmospheric CO_2, plant material, and soil organic matter are depleted in $\delta^{13}C$ relative to the standard and therefore have negative values. In previous work related to the genesis of secondary copper deposits, malachite and azurite were demonstrated to be insensitive to carbon isotope fractionation, making it an ideal tracer for identification of carbon sources[S13,S14]. The $\delta^{13}C$ values measured in our copper(II) carbonates ranged from −22 to −17‰ (Table S3). These values correspond to the typical soil organic carbon $\delta^{13}C$ signature for mixed C3 and C4 plant communities, between −28 and −15‰. Despite the presence of a calcareous bedrock, no significantly heavier $\delta^{13}C$ values resulting from the dissolution of calcareous material in the soil, $CaCO_3 + CO_2 + H_2O \rightleftharpoons Ca^{2+} + 2\,HCO_3^-$, are observed. No stable isotope measurements could be performed at the site owing to its destruction.

Table S3: Measured $\delta^{13}C$ values of mineralized textile fiber samples and modern wool.

**Sample name**	**$\delta^{13}C$ IRMS (‰)**
A1_4A	-22.62
A1_4B	-17.77
A2A3_3A	-17.72
A2A3_3B	-18.84
A1_5	-19.87
S1-S4	-19.42
Wool a	-22.72
Wool b	-24.39

References

(S1) Villard, A. *Creney-Le-Paradis : Prolongement Du Boulevard Georges Pompidou. Rapport de Sauvetage Programmé*; Excavation Report, 1988; p 25.

(S2) Iacconi, C.; Desplanques, E.; Moulherat, C.; L'Héronde, M.; King, A.; Autret, A.; Schoeder, S.; Fayard, B.; Leccia, E.; Bertrand, L. Archaeological Mineralised Textiles from the Iron Age Tumulus of Creney-le-Paradis Support Its Elite Status. *Antiquity* **2024**, *98*, 1306–1320.

(S3) Robbiola, L.; Blengino, J. M.; Fiaud, C. Morphology and Mechanisms of Formation of Natural Patinas on Archaeological Cu–Sn Alloys. *Corros. Sci.* **1998**, *40*, 2083–2111.

(S4) Robbiola, L.; Portier, R. A Global Approach to the Authentication of Ancient Bronzes Based on the Characterization of the Alloy–Patina–Environment System. *J. Cult. Herit.* **2006**, *7*, 1–12.

(S5) Nienhuis, J.; Robbiola, L.; Giuliani, R.; Joosten, I.; Huisman, H.; van Os, B.; Sietsma, J. Curly Malachite on Archaeological Bronze: A Systematic Study of the Shape and Phenomenological Approach of Its Formation Mechanism. *E-Preserv. Sci.* **2016**, *13*, 23–32.

(S6) Hendriks, L.; Caseri, W.; Ferreira, E. S. B.; Scherrer, N. C.; Zumbühl, S.; Küffner, M.; Hajdas, I.; Wacker, L.; Synal, H.-A.; Günther, D. The Ins and Outs of 14C Dating Lead White Paint for Artworks Application. *Anal. Chem.* **2020**, *92*, 7674–7682.

(S7) Messager, C.; Beck, L.; Blamart, D.; Richard, P.; Germain, T.; Batur, K.; Gonzalez, V.; Foy, E. 25 Centuries of Lead White Manufacturing Processes Identified by 13C and 14C Carbon Isotopes. *J. Archaeol. Sci. Rep.* **2022**, *46*, 103685.

(S8) Wacker, L.; Christl, M.; Synal, H. A. Bats: A New Tool for AMS Data Reduction. *Nucl. Instrum. Methods Phys. Res. B* **2010**, *268*, 976–979.

(S9) Ward, G. K.; Wilson, S. R. Procedures for Comparing and Combining Radiocarbon Age Determinations: A Critique. *Archaeometry* **1978**, *20*, 19–31.

(S10) Edinborough, K.; Martineau, R.; Dufraisse, A.; Shennan, S.; Imbeaux, M.; Dumontet, A.; Schauer, P.; Cook, G. A Neolithic Population Model Based on New Radiocarbon Dates from Mining, Funerary and Population Scaled Activity in the Saint-Gond Marshes Region of North East France. *Quat. Int.* **2021**, *586*, 121–132.

(S11) Reimer, P. J.; Austin, W. E. N.; Bard, E.; Bayliss, A.; Blackwell, P. G.; Ramsey, C. B.; Butzin, M.; Cheng, H.; Edwards, R. L.; Friedrich, M.; Grootes, P. M.; Guilderson, T. P.; Hajdas, I.; Heaton, T. J.; Hogg, A. G.; Hughen, K. A.; Kromer, B.; Manning, S. W.; Muscheler, R.; Palmer, J. G.; Pearson, C.; van der Plicht, J.; Reimer, R. W.; Richards, D. A.; Scott, E. M.; Southon, J. R.; Turney, C. S. M.; Wacker, L.; Adolphi, F.; Büntgen, U.; Capano, M.; Fahrni, S. M.; Fogtmann-Schulz, A.; Friedrich, R.; Köhler, P.; Kudsk, S.; Miyake, F.; Olsen, J.; Reinig, F.; Sakamoto, M.; Sookdeo, A.; Talamo, S. The IntCal20 Northern Hemisphere Radiocarbon Age Calibration Curve (0–55 Cal kBP). *Radiocarbon* **2020**, *62*, 725–757.

(S12) Trumbore, S. E.; Druffel, E. R. M. *Role of Nonliving Organic Matter in the Earth's Carbon Cycle*; John Wiley & Sons Ltd: Chichester, UK, 1995; pp 7–22.

(S13) Melchiorre, E. B.; Criss, R. E.; Rose, T. P. Oxygen and Carbon Isotope Study of Natural and Synthetic Malachite. *Econ. Geol.* **1999**, *94*, 245–259.

(S14) Melchiorre, E. B.; Criss, R. E.; Rose, T. P. Oxygen and Carbon Isotope Study of Natural and Synthetic Azurite. *Econ. Geol.* **2000**, *95*, 621–628.
